# Examining Nigerian Undergraduate History Students' Survey Dataset on Gambling Behaviour

**DOI:** 10.3389/fpsyg.2022.944826

**Published:** 2022-07-01

**Authors:** Frances Jumoke Oloidi, Uche Calista Vita-Agundu

**Affiliations:** ^1^Department of History and International Studies, University of Nigeria, Nsukka, Nigeria; ^2^Department of Educational Foundations, University of Nigeria, Nsukka, Nigeria

**Keywords:** dataset, gambling behaviour, history students, missing data, outliers, sampling adequacy

## Introduction

Gambling is a common problem behaviour among Nigerian youth and adolescents (Temitope, 2019; Adebisi et al., 2021; Amazue et al., 2021). Gambling is done both online and offline and most youth perceives it as a business opportunity to get rich overnight (Temitope, 2019; Ede, 2019). Gambling entails risking something valuable with the hope of acquiring something of more value in return (Wilber and Potenza, 2006). Gambling is defined as betting or wagering for money or for materials for a case which has an unknown outcome with an opportunity to make cash or material (Potenza et al., 2002; Lungu, 2020). Gambling behaviour is conceptualised by Ede et al. (2020) as an act of betting with money and other valuables on a game with the tempting intention of gaining unrealistic profits which in turn may result to psychological malfunctioning of individual in the future due to untold losses. Some of the gambling activities found within university environment in Nigeria include but not limited to draft, casino, sport betting, lottery, Baba Ijebu, online gaming, card games among others (Adesina, 2019; Ede, 2019). Gambling is a nationally legalised business and its activities are regulated by government agency of Nigeria known as National Lottery Regulatory Commission. Gambler's behaviour has been identified as having serious consequences on their health and habits, and has been associated with some criminal behaviours such as stealing (Oyebisi et al., 2012; Lavojo et al., 2020). Yet gambling activities have become part and parcel of the normal culture that is practised among different age groups, gender, and socio-economic status (Omanchi and Okpamen, 2018; Temitope, 2019). In Nigeria where gambling is a rapidly growing business, with gambling shops proliferating, gambling has become more rampant among youths even when they are in the school (Ajomale, 2017; Awo et al., 2022).

Gambling behaviour among Nigerian students in higher education institutions has been noted to be a growing public health issue (Oyebisi et al., 2012; Ede et al., 2020). Students' engagement in gambling could be motivated by peer pressure, greed, economic deprivation, personality traits, joblessness and financial strains, which are all shown to be the main catalysts for this behaviour in Nigeria (Binde, 2013; NOIPolls, 2017; Temitope et al., 2019; Lavojo et al., 2020). After adjusting for age, Awo et al. (2022) found that increases in downward counterfactual thinking were related to a decrease in gambling intention, whereas increases in upward counterfactual thinking and impulsivity were linked to an increase in gambling intention among Nigerian students. Moreover, according to a study carried out in Nigeria among university students, the relationship of psychopathic and narcissistic behaviours with gambling increased with age, whereas, at a younger age it was not seen (Onyedire et al., 2021).

However, there is limited publicly available students' gambling behaviour dataset to practically influence future research. Using undergraduate history students' survey dataset, this study examines the sampling adequacy, manages missing data, examines normality, and identifies outliers in the data about their gambling behaviour. A Nigerian undergraduate history student is a student who has been admitted to the Department of History and International Studies at the university; students who enrol in this 4-year program will earn a bachelor's degree in history; they have as a major goal to become proficient historians by the end of the program (University of Lagos, 2020; University of Nigeria, 2020; Oloidi et al., 2022). So, in order to achieve its aim, the programme aims to offer students an understanding of history in a manner that is consistent with the best global practises in university education (Oloidi et al., 2022). The program also seeks to familiarise students with past and present phenomena that have had a significant impact on human beings and that are still making an impact on them (University of Lagos, 2020; University of Nigeria, 2020; Oloidi et al., 2022). Boadu (2015) points out that history is an abstract subject, and, as a result, its learning can be quite challenging for students. In order to easily finance their education, some of these history undergraduates may resort to gambling as an alternative source of income.

Adverts from radio, television, social media, and newspapers have made several types of gambling look enticing and satisfactory to most Nigerian students thereby decreasing the associated stigma with those that plays it regularly (Gbadebo, 2017). Hence, gambling has gained huge social reception and patrons especially among undergraduate students. According to Adesina (2019), an agent from a bet company reported that “60 million Nigerians within the age bracket of 18 and 40 years spend more than N1 billion daily on sports betting.” The report also indicated that a betting company make up to N20 million monthly and spend between N5 million and N7million on winners. Other reports revealed that an increasing inclination toward gambling in Nigeria has some negative effects on students' behaviour and performance in school (Okwaraji et al., 2015; Abayomi et al., 2016). Undergraduate history students who indulge in gambling may develop irrepressible addiction toward it if they are not identified for early intervention. Thus, the objective of the current study was to examine issues related to sampling adequacy, missing data, normality, and outliers in the dataset of Nigerian undergraduate history students' gambling behaviour in order to inform future research and psycho-educational intervention among this population.

## Method

### Participants

The sample size of the participants was 600 undergraduate history students (age range = 18–25 years old; mean age = 22.06 ± 1.50 yrs) selected through multistage sampling procedure. To begin with, two federal universities in Southeast Nigeria were selected using a simple random sampling technique. The second technique used to select history undergraduates from the entire student population of each university was purposive sampling technique. In addition, the snowball sampling technique was used to identify undergraduate history students who were eligible to participate in the survey. Male students comprised 51.3% while female students comprised 48.7% of the total sample. History undergraduates in first year comprised 17.5%, second year students comprised 19.7%, third year students comprised 36.3% and fourth year students comprised 26.5% of the sample. History undergraduates from one out of the two universities studied comprised 49.3% of the sample while the rest were from the second university.

### Material

The instrument used for data collection was a questionnaire titled “Prevalence of Gambling Behaviour Questionnaire (PGBQ)” which was adapted from Ede (2019). The questionnaire has two sections: section “A” dealt on demographic variables such as gender, level of study, and institutions; while section “B” is made up of 23 items questionnaire that extract information on students' gambling behaviours. The items of the instrument were structured in four-point Likert response type as follows: A lot (4), Sometimes (3), A little (2), and Not At all (1). PGBQ was validated by three experts in Faculty of Arts, University of Nigeria.

### Procedure

As part of the data gathering process, the students were sent softcopies of the questionnaire and informed consent form to complete and return within 4 weeks via electronic email. All data collection took place in the month of February of this year. The students were drawn from two public universities that are federally funded in the Southeast of Nigeria.

## Results and Discussion

The dataset from Oloidi and Vita-Agundu's (2022) study which explores the prevalence of gambling was subjected to a test of missing data. Missing data is any response item that the respondents skipped intention or unintentional. The result showed that 11 data sets were missing which represents 1.8% of the dataset. The percentage of the missing data is accepted for survey research design. The data were missing at random (MAR). Pairwise deletion method was used in treating the data because pairwise deletion preserves more information than listwise deletion thereby uses all information observed. This approach is the most suitable for missing at random data because is known to be less biassed (Kang, 2013).

**Figure 1:**
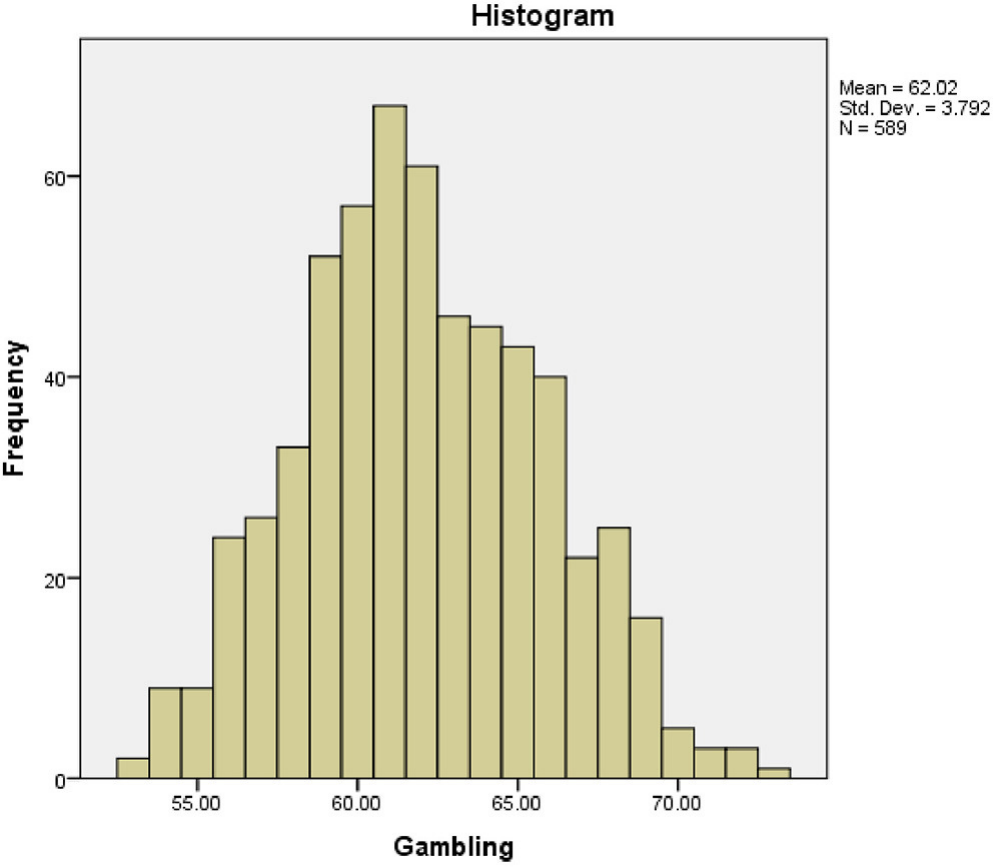
Histogram showing data normality.

The dataset was subjected to normality test using Kolmogorov- Smirnov statistics Normal Q-Q plots, and histogram. The sig-value of Kolmogorov- Smirnov is 0.000 which suggest that assumption of normality is violated based on Lilliefors Significance Correction (Lilliefors, 1967) (statistic = 0.079, df = 589, *p* = 0.000). However, this is expected in a large sample and the sample size of the study is large (*n* = 600). Therefore, histogram was used an alternative to establish the normality test (see Figure 1). The actual shape of the histogram shows that the assumption of normality was not violated. The score appears to be reasonably normally distributed. In addition, the normal Q-Q probability plot indicated that the data is normally distributed since there was a straight line that plots observed value for each score plotted against the expected value from normal distribution.

**Figure 2:**
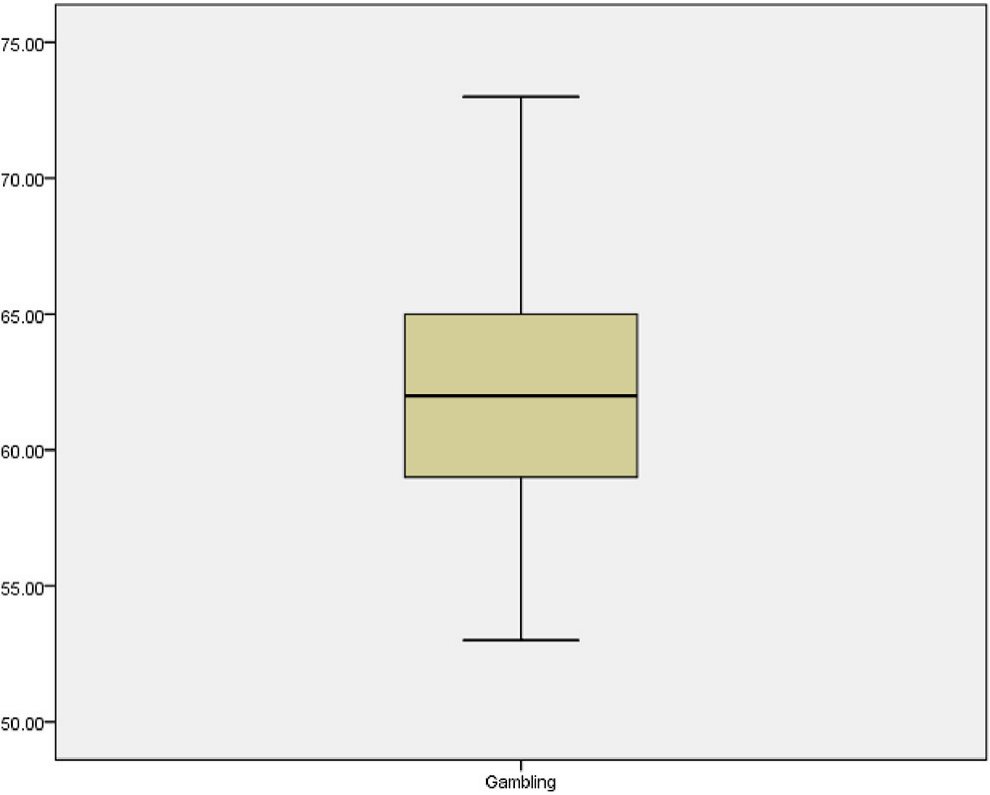
The box plot.

There are several methods of detecting data outliers in a dataset and is dependent on what the dataset is aimed at measuring. According to Aguinis et al. (2013), box plots and scatterplots are used to measure individual variables while Mahalanobis distance is used to measure for multiple variables. Since the dataset of this study is generated from a survey study and meant to explore gambling behaviour of history undergraduates with no intention of comparison or establishing relationship, histogram and boxplots were adopted. First of all, the outlier in our data was examined using histogram, because the sample size of the study is large. The dataset in histogram shows that there is no data point sitting on its own or located at the extreme as indicated by tail of histogram. Hence, the data points were closely clustered together indicating that there was no potential outlier. In addition, second inspection was conducted to explore the existence of outlier in the data using boxplot. The boxplot showed that there was no extreme value extending above 1.5 box lengths from the edge of the box (see Figure 2). Therefore, there is no element of outlier in the data.

This study adopted principal component factor analysis because its mean idea is to reduce the dataset dimension that has much large number of inter-correlated variables as well as retaining as much as possible the variation present in the data set. This reduction was obtained via transforming to a new variable set, the principal components, which are uncorrelated, and which are ordered so that the first few retain most of the variation present in all of the original variables. Using component factor analysis, two preliminary analyses is recommended which include Bartlett's test of sphericity and Kaiser-Meyer-Oikin. Bartlett's Test of Sphericity (Bartlett, 1950) is used to establish that the correlation matrix contains one on the diagonal and zero on the off-diagonal of PGBQ dataset. The Bartlett's Test of Sphericity chi-square coefficient of 0.000 for PGBQ was significant at 0.05 indicating that the assumption of Bartlett's Test of Sphericity was not violated. Therefore, PGBQ data is good to be subjected for factor analysis.

In addition, the measure of sampling adequacy was conducted to confirm whether the sample size of PGBQ dataset is adequate. In this regard, the Kaiser-Meyer-Olkin (KMO; Kaiser, 1974) was adopted because it measures the sampling adequacy based on the ratio of correlations and partial correlations that reflects the extents to which correlations are a function of the variance shared across all variables rather than the variance shared by particular pairs of variables. The sample for the dataset on which the PGBQ was used has KMO value of 0.552 which is within acceptable according to Hoelzle and Meyer (2013) and Lloret et al. (2017).

The extraction technique adopted for this factor analysis was based on Eigen values of 1 which provides the Eigen-decomposition of a matrix for analysis of the matrix structure (Kaiser, 1974; O'Brien, 2007; Williams et al., 2010). It was adopted since it provides clear expression for matrices such as correlation or covariance and vital for finding the minimum or maximum of functions involving metrics (Williams et al., 2010; Watkins, 2018). The numbers of components extracted were nine which explained 50.7% of the variance, respectively. The rotated solution shows that the items of PGBQ showed strong factor loading in nine components; all the items factor loading were above 0.3. Majority of the positive item loaded on component 1–3 while majority of negative items loaded from component four. Few items were complex which could be as result of the reflective nature of the questionnaire items.

## Conclusion

We present the first public dataset on gambling behaviour among a sample of Nigerian history undergraduates in select public universities. We believe it is the first study to also analyze sampling adequacy, missing data, normality, and outliers in undergraduate gambling behaviour data in Nigeria. Data from the study dataset may be useful for further comparative analytic surveys on undergraduate gambling behaviour across academic disciplines in Africa. It may be necessary to examine a multivariate dataset for gambling behaviour among undergraduate history students to shed more light on this problem. The study has some limitations such as that the dataset was only generated with a unidimensional scale, and that it was limited to undergraduate history students. To provide additional insight into this problem behaviour among undergraduate students, future research will have to extend its range of sample by encompassing a more expansive sample of undergraduate students. In future research, the gambling behaviour data should be collected using multi-dimensional scale, which will allow for additional insight into the problem gambling behaviour of undergraduate history students.

## Data Availability Statement

The datasets presented in this study can be found in online repositories. The names of the repository/repositories and accession number(s) can be found in the article/supplementary material.

## Ethics Statement

The studies involving human participants were reviewed and approved by Faculty of Education Research Ethics Committee University of Nigeria Nsukka. The patients/participants provided their written informed consent to participate in this study.

## Author Contributions

FO and UV-A initiated the study, designed the study methodology and conducted the study equally, wrote the initial draft, and revised the manuscript equally. Both authors agree to be accountable for the content of the work, contributed to the article, and approved the submitted version.

## Conflict of Interest

The authors declare that the research was conducted in the absence of any commercial or financial relationships that could be construed as a potential conflict of interest.

## Publisher's Note

All claims expressed in this article are solely those of the authors and do not necessarily represent those of their affiliated organizations, or those of the publisher, the editors and the reviewers. Any product that may be evaluated in this article, or claim that may be made by its manufacturer, is not guaranteed or endorsed by the publisher.

## References

[B1] Abayomi O. Adebayo K. O. Adelufosi A. O. Ibrahim N. O. Mosanya J. T. Suleiman B. T. . (2016). Risky substance use among patrons of gambling venues in Ogbomoso, Oyo State, Nigeria. Int. J. Res. Appl. Natural Soc. Sci. 4, 45–52.

[B2] Adebisi T. Alabi O. Arisukwu O. Asamu F. (2021). Gambling in transition: assessing youth narratives of gambling in Nigeria. J. Gambling Stud. 37, 59–82. 10.1007/s10899-020-09982-x 32996037

[B3] Adesina T. R. (2019). Psychosocial Determinants of Gambling Behaviour among Public Secondary School Students in Osun State, Nigeria. (Doctoral dissertation, Obafemi Awolowo University, Ile-Ife).

[B4] Aguinis H. Gottfredson R. K. Joo H. (2013). Best-practice recommendations for defining, identifying, and handling outliers. Organ. Res. Methods 16, 270–301. 10.1177/1094428112470848

[B5] Ajomale O. (2017). The Rise of the Gambling Culture in Nigeria. The Pulse Newspaper. Retrieved from: https://www.pulse.ng/lifestyle/money/bad-money-habits-the-rise-of-the-gambling-culture-in-nigeria/ce8j4yk (Accessed January 24, 2022)

[B6] Amazue L. O. Awo L. O. Agbo A. A. Ekwe C. N. Ojiaku M. C. (2021). Association of near-miss with two erroneous gambling cognitions and betting intention: evidence from Nigerian adolescents. J. Gambling Stud. 37, 837–852. 10.1007/s10899-020-09994-7 33386515

[B7] Awo L. O. Amazue L. O. Eze V. C. Ekwe C. N. (2022). Mediating role of impulsivity in the contributory roles of upward versus downward counterfactual thinking in youth gambling intention. J. Gambl. Stud. 10.1007/s10899-022-10112-y. [Epub ahead of print]. 35246753

[B8] Bartlett M. S. (1950). Tests of significance in factor analysis. Br. J. Psychol. 3, 77–85. 10.1111/j.2044-8317.1950.tb00285.x

[B9] Binde P. (2013). Why people gamble: a model with five motivational dimensions. Int. Gambl. Stud. 13, 81–97. 10.1080/14459795.2012.712150

[B10] Boadu G. (2015). Effective teaching in history: the perspectives of history student-teachers. Int. J. Humanities Soc. Sci. 3, 38–51.20658077

[B11] Ede M. O. (2019). Effect of group counselling on pathological internet use and gambling among undergraduate students in Federal Colleges of Education in South-East, Nigeria. (Doctoral dissertation), University of Nigeria, Nsukka.

[B12] Ede M. O. Omeje J. C. Ncheke D. C. Agah J. J. Chinweuba N. H. Amoke C. V. (2020). Assessment of the effectiveness of group cognitive behavioural therapy in reducing pathological gambling. J. Gambl. Stud. 36, 1325–1339. 10.1007/s10899-020-09981-y 33037961

[B13] Gbadebo J. (2017). Lawyer to FG: Take Action Against Underage Involvement in Betting. The Nation. Retrieved from: https://thenationonlineng.net/lawyer-to-fgtake-action-against-underage-involvement-in-betting/ (accessed January 14, 2022).

[B14] Hoelzle J. B. Meyer G. J. (2013). “Exploratory factor analysis: basics and beyond,” in Handbook of Psychology: Research Methods in Psychology, eds J. A. Schinka, W. F. Velicer, and I. B. Weiner (Hoboken, NJ: John Wiley & Sons, Inc.), 164–188. 10.1002/9781118133880.hop202006

[B15] Kaiser H. F. (1974). An index of factorial simplicity. Psychometrika 39, 31–36. 10.1007/BF02291575

[B16] Kang H. (2013). The prevention and handling of the missing data. Korean J. Anesthesiol. 64, 402–406. 10.4097/kjae.2013.64.5.402 23741561 PMC3668100

[B17] Lavojo J. A. Bala I. Arogundade A. F. Collins F. S. (2020). Predisposing factors of sport gambling among youths in Taraba State. Sapientia Foundation J. Educ. Sci. Gender Stud. 2, 211–221.

[B18] Lilliefors H. W. (1967). On the Kolmogorov-Smirnov test for normality with mean and variance unknown. J. Am. Stat. Assoc. 62, 399–402. 10.1080/01621459.1967.10482916

[B19] Lloret S. Ferreres A. Hernández A. Tomás I. (2017). El análisis factorial exploratorio de los ítems: análisis guiado según los datos empíricos y el software. Anal. Psicol. 33, 417–432. 10.6018/analesps.33.2.270211

[B20] Lungu C. T. (2020). Gambling among Nigerian youths: implications for counselling. Int. J. Res. Scientific Innov. 7, 179–183.33386515

[B21] NOIPolls. (2017). New Poll Reveals Rising Trend of Gambling in Nigeria. NOI Polls. Retrieved from: https://noi-polls.com/new-poll-reveals-rising-trend-of-gambling-in-nigeria/ (accessed January 12, 2022).

[B22] O'Brien K. (2007). Factor analysis: an overview in the field of measurement. Physiother. Canada 59, 142–155. 10.3138/ptc.59.2.142

[B23] Okwaraji F. E. Aguwa E. N. Onyebueke G. C. Arinze-Onyia S. U. Shiweobi-Eze C. (2015). Gender, age and class in school differences in internet addiction and psychological distress among adolescents in a Nigerian Urban City. Int. Neuropsychiatric Dis. J. 4, 123–131. 10.9734/INDJ/2015/18933

[B24] Oloidi F. J. Sewagegn A. A. Amanambu O. V. Umeano B. C. Ilechukwu L. C. (2022). Academic burnout among undergraduate history students: effect of an intervention. Medicine 101, e28886. 10.1097/MD.0000000000028886 35363205 PMC9282037

[B25] Oloidi F. J. Vita-Agundu U. C. (2022). Prevalence of gambling behaviour among undergraduate history students. (Unpublished Manuscript). Available online at: https://www.researchgate.net/publication/361099419_Prevalence_of_gambling_behavour_among_undergraduate_history_students (accessed January 24, 2022).

[B26] Omanchi S. A. Okpamen K. O. (2018). The changing patterns of gambling in Benue state: the case of emerging role of ICT (Information and Communications Technology) in Contemporary Makurdi Metropolis. AASCIT Commun. 5, 29–36.

[B27] Onyedire N. G. Chukwuorji J. C. Orjiakor T. C. Onu D. U. Aneke C. I. Ifeagwazi C. M. (2021). Associations of Dark Triad traits and problem gambling: moderating role of age among university students. Curr. Psychol. 40, 2083–2094. 10.1007/s12144-018-0093-3

[B28] Oyebisi E. O. Alao K. A. Popoola B. I. (2012). Gambling behaviour of university students in South-Western Nigeria. IFE Psychol. Int. J. 20, 252–262.

[B29] Potenza M. N. Fiellin D. A. Heninger G. R. Rounsaville B. J. Mazure C. M. (2002). Gambling. J. General Internal Med. 17, 721–732. 10.1046/j.1525-1497.2002.10812.x 12220370 PMC1495100

[B30] Temitope B. E. (2019). Patterns and prevalence of gambling behaviour among youths in south-west Nigeria: a case study of youths in Oyo and Ekiti State. Br. J. Psychol. Res. 7, 22–46.

[B31] Temitope B. E. Oyekola A. Mary B. A. (2019). Personality traits and financial strain as determinants of gambling behaviour among youth in Nigeria: a case study of youths in Oyo State and Ekiti State. Am. Int. J. Soc. Sci. Res. 4, 1–8. 10.46281/aijssr.v4i1.235

[B32] University of Lagos (2020). History & Strategic Studies. Retrieved from: https://unilag.edu.ng/?page_id=2692 (accessed December 15, 2021).

[B33] University of Nigeria (2020). History and international studies. Retrieved from: https://www.unn.edu.ng/academics/faculties/arts/history-and-international-studies/ (accessed December 15, 2021).

[B34] Watkins M. W. (2018). Exploratory factor analysis: a guide to best practice. J. Black Psychol. 44, 219–246. 10.1177/0095798418771807

[B35] Wilber M. K. Potenza M. N. (2006). Adolescent gambling: research and clinical implications. Psychiatry (Edgmont). 3, 40–48. 20877546 PMC2945873

[B36] Williams B. Onsman A. Brown T. (2010). Exploratory factor analysis: a five-step guide for novices. Austral. J. Paramed. 8, 990399. 10.33151/ajp.8.3.93

